# The Planet Mars

**Published:** 1883-09

**Authors:** 


### THE PLANET MARS.

Professor Lockyer is of the opinion that human life on the planet Mars may be very much like human life on the earth ; the light cannot be so bright, but the organs of sight may be so much more susceptible as to make the vision quite as good. The heat is probably less, as the polar snows certainly extend further, but by no means less in proportion to the lessened power of the solar rays. The professor agrees with others, that several remarkable seas—including inland seas, some of them connected and some not connected by straits with still larger seas—are now definable in the southern hemisphere, in which, as is the case also with the earth, water seems to be much more widely spread than in the northern hemisphere. There is, for example, a southern sea exceedingly like the Baltic in shape ; and there is another and still more remarkable sea, now defined by the observations of many astronomers—one near the equator, a long straggling arm twisting almost in the shape of an S laid on its back, from east to west, at least 1,000 miles in length and 400 in breadth.

### THE CITY OF CAIRO.

The city of Cairo is the modern capital of Egypt. It is built on the plain and the lower slopes of a rocky range, the citadel built by Saladin standing two hundred and fifty feet above the level of the town. The houses of the rich class are built in an elaborate arabesque style, the windows shaded with projected cornices of graceful woodwork and ornamented with stained glass. Among the buildings which owe their existence to European influence, the Italian opera, the French theatre, the Hippodrome, and the French and English churches may be mentioned. The system of public instruction is surpassingly good. The population of Cairo is 350,000, equal to that of St. Louis.

THERE is no man who has so little spare time as the one who is thoroughly idle. Idling is of itself a business which finds even all the waking hours of the day not quite sufficient for its needs.

### "FOOLSCAP."

The story may or may not be true that King James I. of England *knighted* a chine of beef that pleased his palate particularly well, and so immortalized the name "Sir-loin." But this is only one of a hundred nouns in common use whose history is equally whimsical.

Everybody knows what foolscap paper is, but we doubt whether one in a hundred of those who use it can tell why it is so called.

When Oliver Cromwell became Protector of England, he caused the stamp of the Cap of Liberty to be placed upon the paper used by the government. Soon after the restoration of Charles II., when he had occasion to use some paper for dispatches, some of this government paper was brought to him. On looking at if he inquired the meaning of it, and on being told, he said, "Take it away; I'll have nothing to do with a fool's cap."

Thus originated the word *foolscap*, which has since been given to a size of writing-paper usually about sixteen by twenty-three inches.

